# Supplementary material for: 60 million years of glaciation in the Transantarctic Mountains
Source: Nat Commun. 2022 Sep 21;13:5526. doi: 10.1038/s41467-022-33310-z (PMC9492669; doi:10.1038/s41467-022-33310-z)
Supplement: Supplementary file 1 — Supplementary Information [file 41467_2022_33310_MOESM1_ESM.pdf]

‘60 million years of glaciation in the Transantarctic Mountains’

Supplementary information

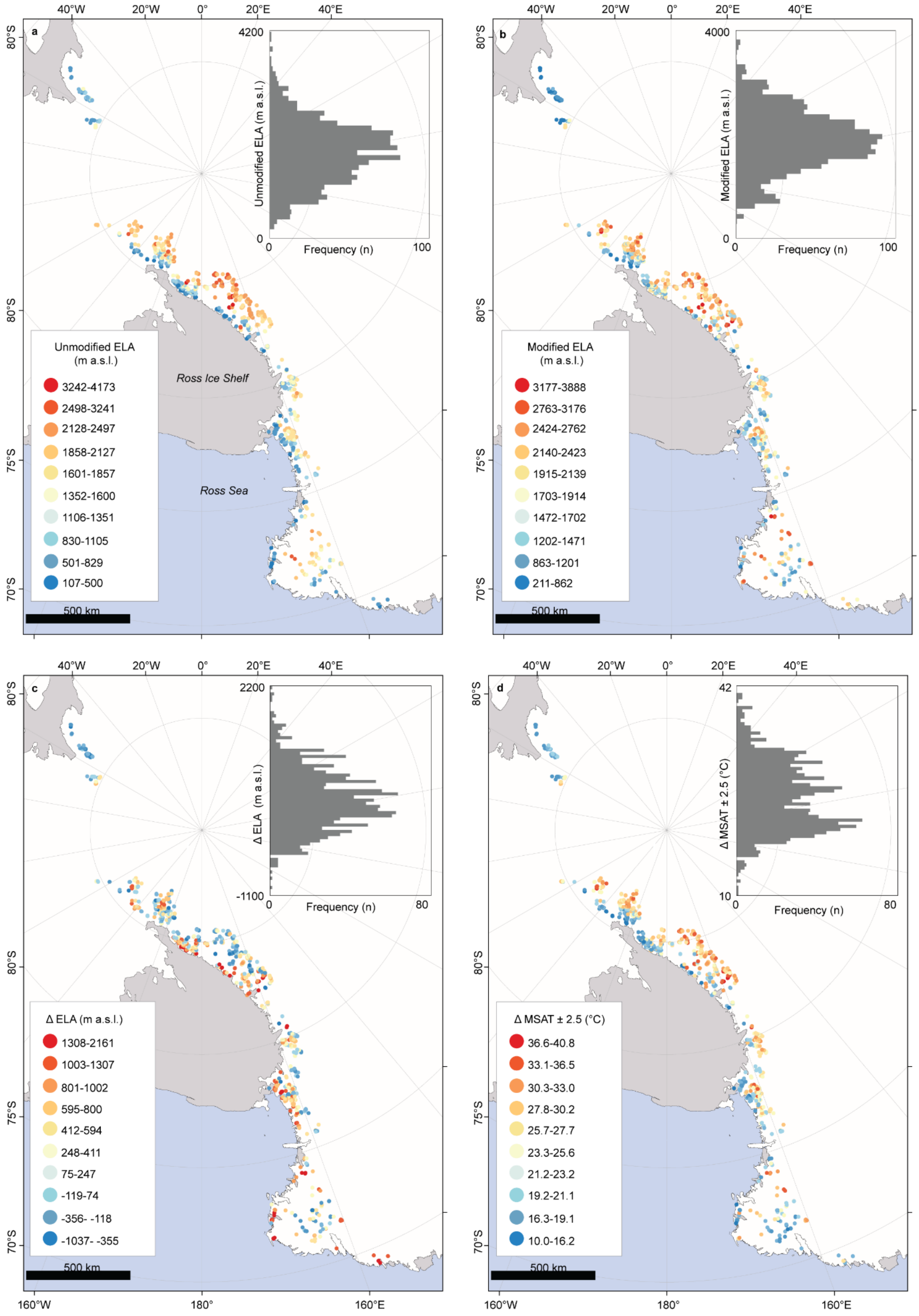

Supplementary Fig 1. **Glacier-free cirques in the Transantarctic Mountains.** (a) Cirques coloured according to their unmodified mountain glacier equilibrium line altitudes (ELAs) – i.e. based on each cirque's minimum and maximum altitude and a THAR of 0.35. (b) Cirques coloured according to their ELAs when modified to 'median' topography at the Eocene-Oligocene boundary (34 Ma)[46]. (c) Cirques coloured according to the altitude difference between modified and unmodified ELAs (ΔELA). (d) Cirques coloured according to the increase in mean summer temperature (ΔMSAT), relative to present, required for them to be occupied by temperate (warm-based) mountain glaciers. In each panel, the inset histogram shows the frequency distribution of values. Antarctic coastline data from the SCAR Antarctic Digital Database, accessed [2021][36] license: https://creativecommons.org/licenses/by/4.0/

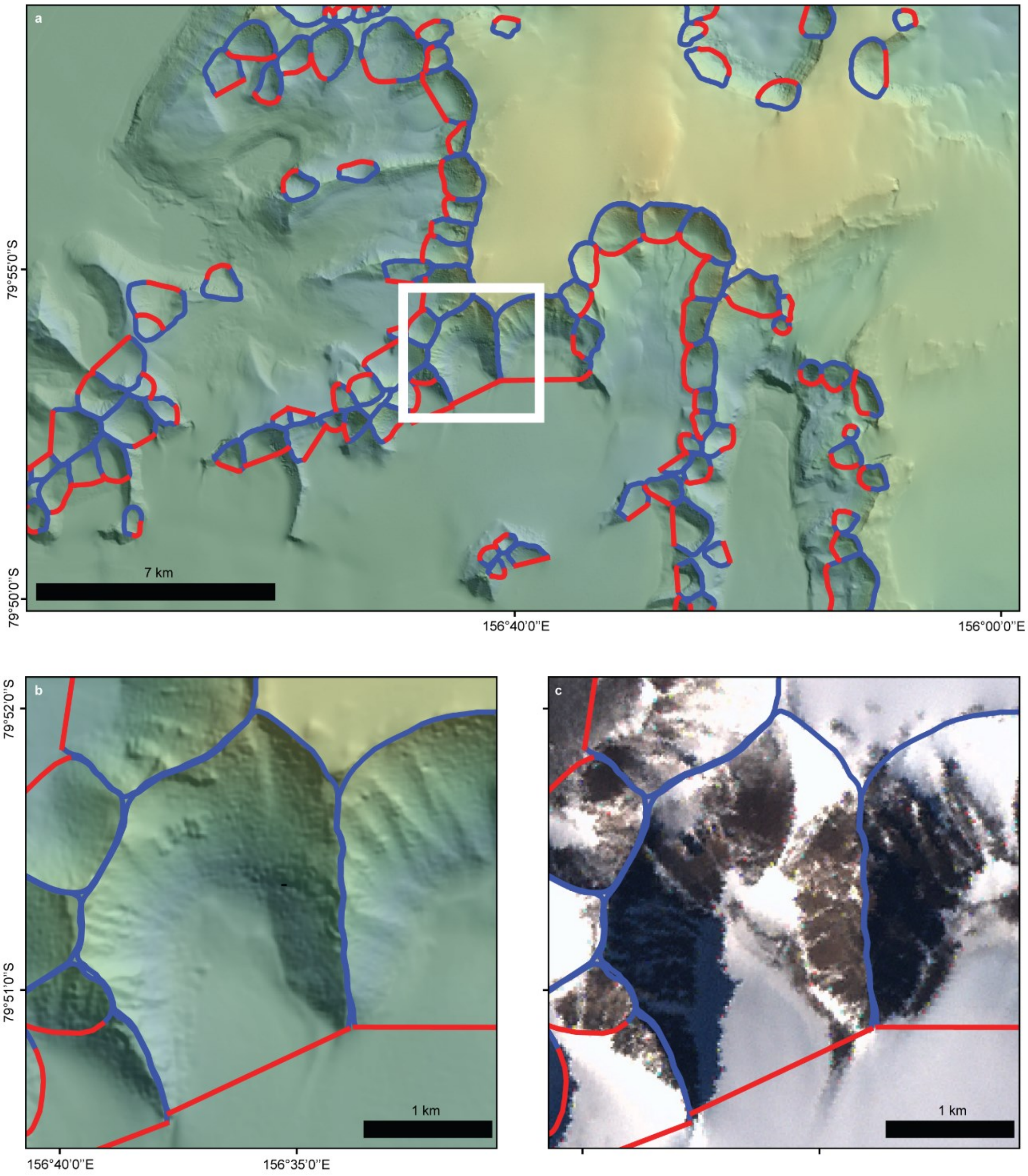

Supplementary Fig 2. **Examples of mapped cirques in the Dry Valleys (part of the Transantarctic Mountains)**. Blue outlines represent cirques, red lines are their thresholds (see Methods for details). Note: not all of these cirques are classified as 'glacier-free'. The white rectangle in (a) shows the region of focus in (b) and (c). In (a) and (b), the background shows the REMA DSM. In (c), the background shows a Landsat 7 ETM+ satellite image.

Supplementary Table 1. Published estimates of Antarctic sea level palaeo mean summertime air temperatures (MSATs) used in the present study.

**Period**	**MSAT (°C)**	**Location**	**Proxy**	**Source**
Late Palaeocene (60–56 Ma)	25.7 ± 2.7	Antarctic Peninsula	Fossil Plants	[22]
Early Eocene (56–48 Ma)	~23	Antarctic Peninsula	Fossil Plants	[23]
Middle Eocene (48–40 Ma)	24 ± 2.7	Antarctic Peninsula	Fossil Plants	[22]
Oligocene (34–23 Ma)	4–12	Ross Sea (offshore from Cape Roberts, TAM)	Fossil Plants	[27–29]
Mid Miocene Climatic Optimum (15 Ma)	7 ± 4	Ross Sea (McMurdo Sound, offshore from TAM)	Pollen, leaf-wax hydrogen isotopes, and model simulations	[30,31]

Supplementary Table 2. Published estimates of Antarctic mean annual palaeo precipitation (ppt) referred to in the present study.

**Period**	**ppt (mm $^{a-1}$)**	**Location**	**Proxy**	**Source**
Late Palaeocene (60–56 Ma)	~2100	Antarctic Peninsula	Fossil Plants	[22]
Middle Eocene (48–40 Ma)	~1534	Antarctic Peninsula	Fossil Plants	[22]
Late Eocene (40–34 Ma)	~1000	Ross Sea (offshore from Cape Roberts, TAM)	Sediment geochemistry	[19]
Oligocene (34–23 Ma)	500–800	Ross Sea (offshore from Cape Roberts, TAM)	Sediment geochemistry	[19]
Mid Miocene Climatic Optimum (~15 Ma)	~600	Ross Sea (offshore from Cape Roberts, TAM)	Sediment geochemistry	[19]
~13.96 Ma	150	Dry Valleys	Vegetation comparison with present-day James Ross and Vega Islands	[19, 32]

Supplementary Table 3. Mean summer air temperatures (MSAT) at the equilibrium line altitudes (ELAs) of a range of present-day temperate glaciers[21]. Note: not all of these glaciers are cirque-type, some are outlets of icefields and some are valley glaciers.

**Glacier**	**Lat**	**Lon**	**MSAT @ ELA (ERA-Interim, °C)**
Chacaltaya	16°21′S	68°07′W	-3.69
Rikha Samba	28°49′N	83°30′E	-1.30
Qiyi	39°23′N	96°59′E	-0.64
EB050 (E09)	27°58′N	86°46′E	-0.26
No. 1 Glacier Urumqi	43°07′N	86°49′E	-0.04
Abramov	39°38′N	71°36′E	0.85
Hodges Glacier	54°17′S	36°30′W	1.06
Shuiguanhe No. 4	37°33′N	101°45′E	1.11
Findelengletscher	46°00′N	7°52′E	1.31
Vernagtferner	46°52′N	10°49′E	1.41
Martial Este	54°47′S	68°24′W	1.48
Tsentralniy Tuyuksuyskiy	43°03′N	77°05′E	1.51
Careser	46°27′N	10°41′E	1.57
Hintereisferner	46°48′N	10°46′E	1.90
Glaciar de Los Tres	49°20′S	73°00′W	1.92
Pasterze	47°06′N	12°42′E	1.97
Wurten Kees	47°02′N	13°00′E	2.11
Goldberg Kees	47°02′N	12°58′E	2.12
Kleinfleiss Kees	47°03′N	12°57′E	2.27
Griesgletscher	46°26′N	8°20′E	2.70
Basodino	46°25′N	8°29′E	2.79
Ram River Glacier	51°51′N	116°11′W	2.95
Aletschgletscher	46°30′N	8°02′E	3.10
Rhonegletscher	46°37′N	8°24′E	3.17
Sonnblick Kees	47°07′N	12°36′E	3.46
Minaret	58°53′N	63°41′W	3.69
Argentiere	45°57′N	6°59′E	3.80
Peyto Glacier	51°40′N	116°33′W	3.82
Yuri Glacier	56°58′N	130°42′W	3.96
Claridenfirn	46°51′N	8°54′E	4.00
Maladeta	42°39′N	0°38′E	4.26
Nisqually Glacier	46°08′N	121°44′W	4.28
Alexander Glacier	57°06′N	130°49′W	4.44
Dzankuat	43°12′N	42°44′E	4.53
Hidden	58°56′N	63°33′W	4.57
Abraham	58°56′N	63°32′W	4.73
Zavisha Glacier	50°48′N	123°25′W	4.90
Superguksoak	58°57′N	63°47′W	4.94
Place Glacier	50°16′N	122°36′W	5.21
Bridge Glacier	50°49′N	123°33′W	5.39
Sykora Glacier	50°53′N	123°34′W	5.46
Tasman Glaceir	43°30′S	170°20′E	5.62
Andrei Glacier	56°57′N	130°59′W	5.74
Helm Glacier	49°58′N	123°00′W	5.98
Tiedemann Glacier	51°20′N	125°00′W	6.27

Marmolada	44°07′N	7°23′E	6.28
Calderone	42°28′N	13°37′E	6.76
Bench Glacier	51°27′N	124°56′W	6.80
Woolsey Glacier	51°01′N	118°12′W	7.03
Lemon Creek Glacier	58°23′N	134°14′W	7.48
Sentinel Glacier	49°54′N	122°59′W	7.51
South Cascade Glacier	48°45′N	121°03′W	8.02
Taku	58°33′N	134°08′W	8.75

Supplementary Discussion 1:Sensitivity to input topography

A key element of this study is the modification of ELAs using the elevation difference between the present-day and relevant palaeo topography. In the manuscript we report values based on the ‘median’ reconstruction of topography at 34 Ma (given the focus on conditions prior to widespread ice-sheet development in Antarctica), but here consider how results vary when ELAs are modified to nine different reconstructions of palaeo topography (i.e. ‘minimum’, ‘median’ and ‘maximum’ reconstructions of topography at 34 Ma, 23 Ma, and 14 Ma)[46]. Supplementary Fig 3a shows the ELAs modified to different topographies, and Supplementary Fig 3b shows ELA differences relative to unmodified values (i.e. those based on present-day topography). This comparison illustrates differences between the datasets. In most cases, modification to palaeo topographies results in an increase in ELAs (supplementary Fig 3b). One exception is when the 34 Ma ‘minimum’ topography is considered, which results in notably lower ELAs that the other datasets and ELAs generally below unmodified values (supplementary Fig 3). Supplementary Fig 4 shows resulting impacts on how cirques are classified (i.e. whether glacier-free, occupied by temperate or cold-based ice) at different time periods. This illustrates that choice of topography has limited impact overall – i.e. there is consistent evidence that a small number of cirques in the TAM were occupied by temperate mountain glaciers during the Late Palaeocene (~66–56 Ma). By the Middle Eocene (~48–40 Ma) glaciation was more widespread, with temperate mountain glaciers in a number of cirques. During the Late Eocene (40–34 Ma) temperate mountain glaciation was widespread, and during the Oligocene (~34–23 Ma) and Mid Miocene

Climatic Optimum (~15 Ma), every TAM cirque was occupied by glacial ice. Again, the one exception to the above is when ELAs are modified to the 'minimum' reconstruction of topography at 34 Ma. In this case, there is no evidence of glaciation of the TAM during the Late Palaeocene or Middle Eocene, and glaciers likely first formed during the Late Eocene. This reconstruction also indicates that most cirques in the TAM were ice-occupied during the Oligocene and Mid Miocene Climatic Optimum but (in contrast to results based on other topographies) that the majority (~80% and 71%, respectively) were occupied by temperate mountain glaciers (Supplementary Fig 4). These contrasts between results based on on the 'minimum' 34 Ma topography and all other topographies reflect the fact that reconstructed elevations in the TAM are conservatively low in the 'minimum' 34 Ma topography to allow for the unresolved uncertainty (and debate) regarding the magnitude of any post-34 Ma tectonic uplift along the mountain range[46].

Finally, supplementary Fig 3b indicates that during the past ~34 Ma, in most cases, glacier-free cirques in the TAM were located at higher elevations than at present. However, as noted in the Methods section, it is unlikely that (in terms of size and shape) the cirques themselves were modified extensively over this period. This might indicate that a substantial portion of this elevation change was driven by ice sheet loading/unloading, rather than by cirque-focused erosion.

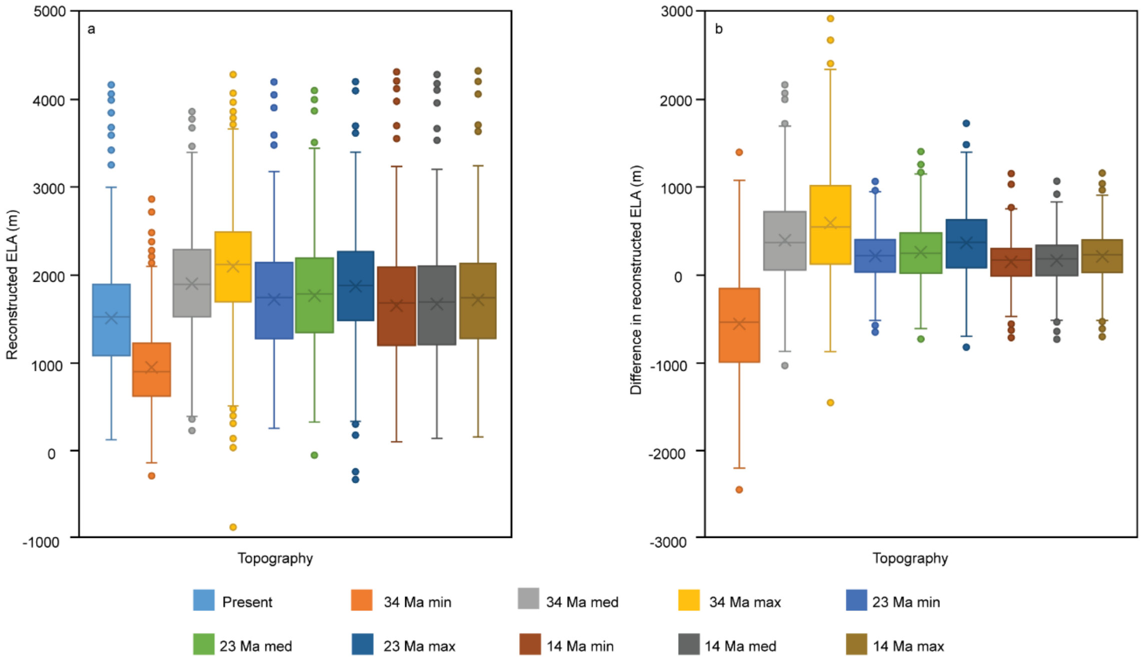

Supplementary Fig 3. **Reconstructed equilibrium line altitudes (ELAs) derived from all (n = 1,292) glacier-free cirques in the Transantarctic Mountains.** (a) ELAs when modified to 'minimum', 'median', and 'maximum' reconstructions of topography at 34 Ma, 23 Ma, and14 Ma[46]. (b) Differences in ELAs relative to unmodified values (i.e. those based on present-day topography). In both (a) and (b) boxplots show the median (horizontal line), mean (cross), 1st and 3rd quartiles, and outliers (i.e. values more than 1.5 box lengths beyond the interquartile range).

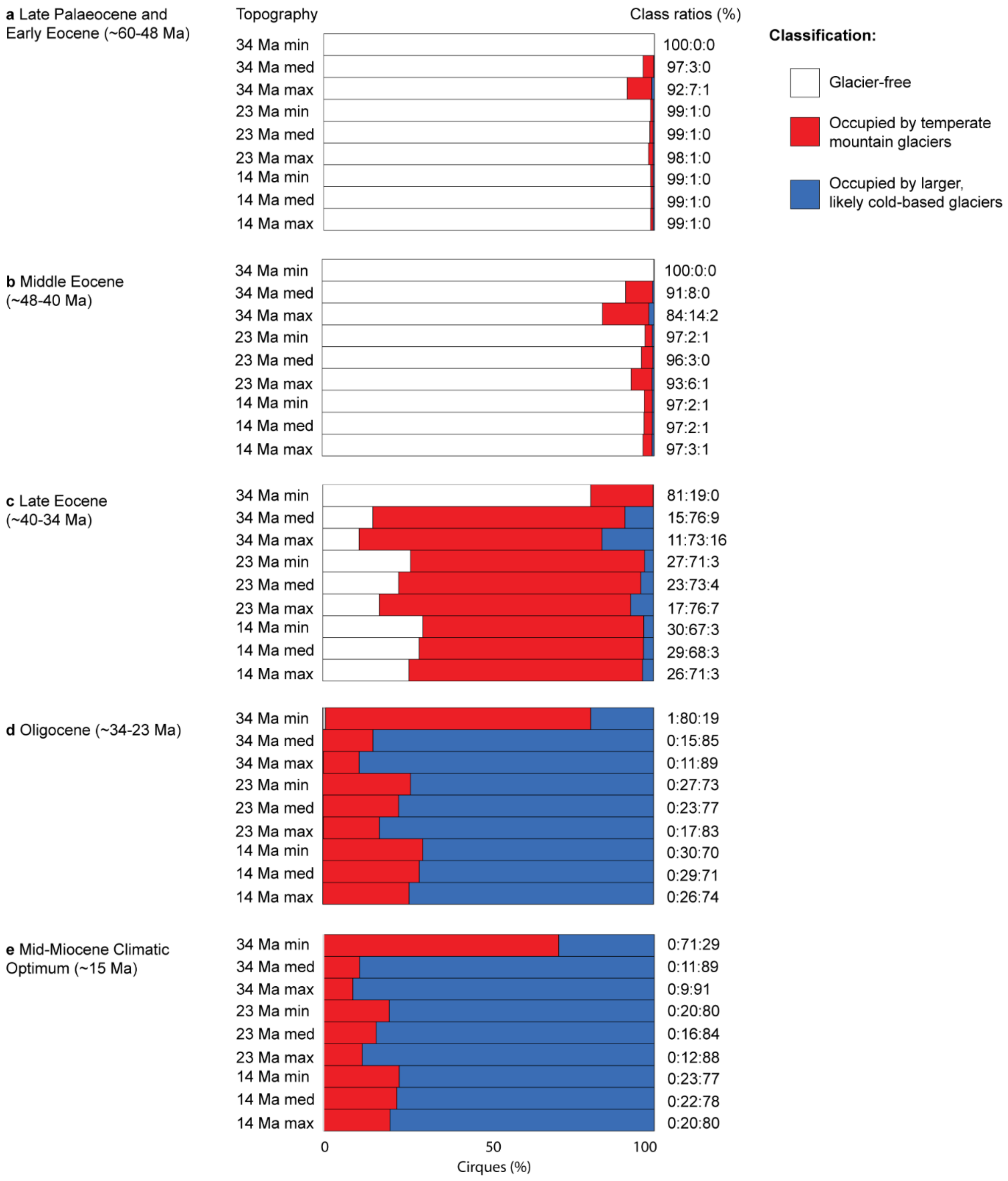

Supplementary Fig 4. **All (n = 1,292) glacier-free cirques in the Transantarctic Mountains classified according to their glacial status during various periods of the Cenozoic (a–e), showing the variability arising from the use of different topographies to calculate former ELAs**. Here we consider ‘minimum’, ‘median’ and ‘maximum’ reconstructions of topography at 34 Ma, 23 Ma, and 14 Ma[46].
