# Supplementary material for: 60 million years of glaciation in the Transantarctic Mountains
Source: Nat Commun. 2022 Sep 21;13:5526. doi: 10.1038/s41467-022-33310-z (PMC9492669; doi:10.1038/s41467-022-33310-z)
Supplement: Supplementary file 3 — Description of Additional Supplementary Files [file 41467_2022_33310_MOESM3_ESM.pdf]

File name: Supplementary Data 1

Description: ESRI shapefile of all cirques mapped in the Transantarctic Mountains.

File name: Supplementary Data 2

Description: Keyhole Mark-up Language file of all cirques mapped in the Transantarctic Mountains.

File name: Supplementary Data 3

Description: ESRI shapefile of all glacier-free cirques mapped in the Transantarctic Mountains.

File name: Supplementary Data 4

Description: Keyhole Mark-up Language file of all glacier-free cirques mapped in the Transantarctic Mountains.

File name: Supplementary Data 5

Description: ESRI shapefile of the thresholds of all cirques mapped in the Transantarctic Mountains.

File name: Supplementary Data 6

Description: Keyhole Mark-up Language file of the thresholds of all cirques mapped in the Transantarctic Mountains.

File name: Supplementary Data 7

Description: Spreadsheet of the attribute data for all glacier-free cirques mapped in the Transantarctic Mountains.
